# Report of Surgical Cases Treated at the Battles of Pea Ridge and Prairie Grove, Ark.

**Published:** 1863-06

**Authors:** E. A. Clark

**Affiliations:** Surgeon 8th Missouri Volunteer Cavalry


### CHICAGO MEDICAL JOURNAL.

VOL. VI.] JUNE, 1863. [No. 6.

### ORIGINAL COMMUNICATIONS.

REPORT OF SURGICAL CASES TREATED AT THE BATTLES OF PEA RIDGE AND PRAIRIE GROVE, ARK.

### PRIMARY AND SECONDARY AMPUTATIONS—RESECTIONS—EXSECTIONS, &c., &c.

By E. A. CLARK, M. D., Surgeon 8th Mo. Vol. Cavalry.

It is hoped that surgeons who treat their cases in private practice, with all the salutary influences of home, a pure atmosphere, careful nursing and a correct system of hygiene, will not be startled at the great mortality of cases—seemingly the most mild—treated in the army, under all the disadvantages we have to encounter in the field, where, under ordinary circumstances, the most necessary comforts of life are not available. Besides, during an engagement of any importance, the number of surgeons is never adequate to even give the wounded a casual examination, and apply temporary dressing to those requiring it, fast as they are carried from the field. When it is, especially at this time, that the most serious wounds should be carefully examined—and where at all necessary—all capital operations performed. But upon this point even some army surgeons take issue, and spin fine theories in favor of secondary operations. But thus far, I have heard this opinion maintained mostly amongst those who have had little or no experience in military surgery. When, I think, every surgeon in the army who has had an opportunity of judging, must admit, that, in the great majority of cases, where amputation will probably be required, that it should be done immediately, before re-action is established; for in that peculiar condition of concussion immediately following a wound of such severity, the nervous system seems, in a measure, anæsthetized, and the patient will succumb much less to the operation, than at any reasonable period after the injury. Besides, it will be observed that re-action from both the wound and the amputation will be quite as readily established (except, perhaps, it be high up in the thigh) as it will after the secondary operation. In evidence of these facts, I have collected a number of cases to present which occurred at the battles of Pea Ridge and Prairie Grove, Arkansas.

At the time of the former engagement, on the 6th, 7th and 8th of March, 1862, I was connected with an Illinois regiment, and had under my immediate charge all the wounded of one brigade, amounting to 227; and there being an unusual scarcity of medical officers—no regiment having to exceed one—of course, it required all my time during the engagement to apply temporary dressings as the wounded came from the field. However, in a number of cases of wounds of the lower extremities, where the bones were badly comminuted, I amputated at once on the field. Of these, there were six cases of badly comminuted fracture of the tibia and fibula, from gunshot wounds, occurring in men of an average constitution, of those subsequently amputated. Also four cases of comminuted fracture of the femur, rendering amputation necessary at the middle third. Of those remaining, there were five with fracture of the leg at about the same point; and three of the thigh, though less comminuted than the others, so as to indicate a probability, by conservative treatment, of restoring a union in the fractures. But afterwards deeming it expedient to perform the secondary operation, some weeks after the date of the injury—the comparative results were as follows: The six cases of primary amputation of the leg, all recovered without any remarkable symptoms, except in one case, in which secondary hæmorrhage appeared some five days after the operation, but with no evil results. Of the three cases of primary amputation of the femur two recovered, and one died four weeks after the operation from typhoid fever, but with the stump in good condition. Of the five cases of secondary amputation of the leg all died but one, and for quite a time I had almost despaired of saving him, but by the most diligent attention and his excellent constitution, he survived the fate of the others. Of the three secondary amputations at the femur all died. In all these cases of secondary amputation, sloughing of the stump and early prostration were the characteristic symptoms in every case, and in two cases of amputation at the leg, and one of the thigh—after the sloughing of the stumps had been considerable—secondary hæmorrhage ensued of an alarming nature, from the too early separation of the ligatures, and which required a second tying of the vessels, but without a very considerable loss of blood. In all these cases the secondary operation had the decided advantage. First, the wounds were not so severe, and the bones much less comminuted; inflicting a much less shock to the system; and at the time these cases were operated upon, they were in a comfortable—or rather comfortable—hospital, where at least they could have good attention and careful nursing, with a generous diet. While those operated upon first, were lying out of doors, in quite cold weather, for two days and nights with scarcely anything to eat, and but little medical attention, then were carried twenty miles in government wagons, to Cassville, Mo.

During the time of the engagement at Prairie Grove, on the 7th Dec., 1862, the opportunity for performing primary operations was less favorable than at Pea Ridge, as all the wounded, amounting to 900, fell in the course of five hours, the medical officers had but time to place them in a comfortable position and apply such immediate dressings as they demanded; and being ordered the next morning to remove all the wounded without delay to Fayetteville, a distance of ten miles, which deprived the surgeons of performing such operations as seemed indeed imperative. However, in the field hospital of which I had charge, I performed two amputations at the middle of the thigh, for severely comminuted fracture of the femur, immediately after the wounds were received, before re-action was established, and while the patients were very much depressed from the concussion; both of these cases recovered without any sloughing of the flaps, or secondary hæmorrhage. In addition to these, I amputated at the same time three cases of comminuted fracture of the tibia, immediately below the knee joint, with equally favorable results, no untoward symptoms occurred and all had a speedy recovery. Of those who came under my immediate observation in the general hospital, at Fayetteville, where the operations were deferred until suppuration was established in the wounds and the constitution somewhat depressed, twelve cases of fractured femur were amputated at the middle and lower third of the thigh, under circumstances much more favorable than those amputated upon the field; yet, but one out of the twelve recovered. In every case, the flaps sloughed away so as to leave the bones denuded, and in five of them, secondary hæmorrhage ensued from sloughing of the vessels, so as to require a second ligation. One died from secondary hæmorrhage, and the remainder from prostration consequent upon repeated attacks of hæmorrhage, excessive sloughing, and suppuration of the stumps. In the case that recovered, the flaps entirely sloughed away, leaving about one inch and a half of the bone exposed, though not denuded of its periosteum, and by cutting away a margin of the granulations around the bone and applying four strips of adhesive plaster twelve inches long and two inches in width, extending six inches above the edge of the stump, and attaching their free ends together, so as to apply extension over the foot of the bed, drawing the muscles forward so that granulations were soon thrown out over the end of the bone, and the patient recovered with a good stump.

Besides these cases of secondary amputation, there were eight of the leg operated upon at the same time, four of them at the upper third near the knee joint, three at the middle third and one at the lower third of the tibia, all of which were comminuted fractures of both bones, three of which had become gangrenous below the wounds at the time of the operation. But out of the eight, only two recovered, and they were of those amputated at the middle and lower third. In all these cases the flaps sloughed very early, and in two of those amputated at the middle third secondary hæmorrhage appeared on the fourth day, but was controlled by pressure and styptics. These cases resulted fatally from the same causes as those above mentioned of the thigh. These were all the cases that were under my control, and of which I kept any notes. But these results compare with the statements of all the surgeons in charge of the different wards in the city. However, I believe none performed any amputations upon the field, so as to make any data for a comparison between the primary and secondary operation. It is true, the few cases that have come under my observation, have not been sufficient to establish to a very great extent the proper practice in these cases, or prove very clearly this important principle in military surgery. But so far as they go, the comparative difference in the results of these cases, as I have stated them, are such as would be likely to influence the opinion of the surgeon under whose observation they occurred. And had the cases been hundreds, doubtless the comparative results would have been the same; and quite sufficient to influence the future practice of a proper observer.

The flap and circular operation performed in those cases of amputation at the thigh, seemed to possess but little advantage over each other. All the cases of secondary hæmorrhage occurred in the flap operation, from sloughing of the flap and artery, where the vessel was ligated in the flap; but in the circular operation, where the artery was retracted in the base of the stump, this accident did not occur, though the same amount of sloughing in the circular operation, of course, would leave a greater length of bone denuded—and, in this respect, the flap operation had the advantage.

The great proportion of mortality in all these cases would seem to indicate the necessity for a more conservative system of surgery, by properly adjusting the fracture with the most convenient apparatus, and by a judicious medication and diet, trusting the remainder of the case to nature. But even the results of this practice has not proven to be "conservative surgery," so far as my experience in the treatment of such cases has convinced me. After the battle of Pea Ridge, I selected from the hospital in my charge eight cases of fractured femurs, such as were the least comminuted, and indicated the most favorable prognosis. These cases were treated with different kinds of apparatus, owing to the peculiarity of the displacement of the fracture in each particular case. In one case, where there seemed to be no disposition to contraction of the muscles or sliding of the fragments, I dressed with a starch bandage, which seemed to keep the fragments in perfect apposition, and prevent the least mobility in the limb—this bandage, of course, had traps cut in it, so as to allow the pus to escape and the wound to be dressed; but union failed and the patient, after six months' suffering, died of debility and prostration. In all the other cases there was considerable contraction of the muscles with displacement of the fragments; two of them were dressed with Physic's splint, modified by the adhesive strips instead of the slipper; and instead of the perineal band, applied two long strips of adhesive plaster one on the front and the other on the posterior aspect of the thigh, extending up, and passing through the mortice in the upper end of the splint. This means of counter extension, I found much preferable to the old method of applying this splint; it obviates that very extensive excoriation of the perineum, which in protracted cases is such an unpleasant consequence in the use of the perineal band. But these cases, too, failed to unite, and the patients died, one in four and the other in five months from the date of injury. In the remaining five cases, I applied moderate extension by means of adhesive strips applied to the leg, as in the dressing for Physic's splint. To the end of these strips was attached a cord, passing through a pulley fixed on the foot of the bed, with a weight attached proportionate to the amount of extension required, and the foot of the bed elevated from six to eight inches so as to prevent the patient sliding down in the bed. This dressing I found the most convenient and pleasant to the patient, the limb rarely requiring any other attention than merely to keep its under surface upon the same plane with the line of extension. But in all these cases—save one—union failed—abscesses formed—as in the other cases—between the muscles. the ends of the fragments became glazed with cartilage, and, in from three to five months the patients died of hectic and prostration. The one who recovered after a tedious confinement of sixteen weeks, finally got up with a firm union in the fracture and a shortening of but one inch.

In addition to these cases, I had also in the same ward six cases of compound fracture of the tibia and fibula, four at the middle and two at the lower third. In all of these cases the bones were very much comminuted, but being more superficial, I removed all the loose fragments of bone and applied the starch bandage soon as the swelling had sufficiently subsided, then allowed the patients to exercise on crutches soon as the dressing had become fixed. All of these cases recovered except one, who from a scrofulous habit and a cachectic condition of the constitution at the time of injury—union failed and the patient died in six weeks from hectic.

Bearing in mind the result of the cases of fractured femur at the battle of Pea Ridge, I had decided upon a different course of treatment, should I again have a similar class of cases. After the battle of Prairie Grove, in the ward to which I was assigned in the General Hospital at Fayetteville, besides those amputated, I had nine cases of compound fracture of the femur from musket balls, and all of which were more or less comminuted—equally so with those just described. One of these—the least comminuted—I treated on Smith's wire splint, as described by him in one of last year's numbers of the *American Journal of Medical Science*—without any interference whatever with the wound or fracture, and with a perfect success, it being also very pleasant to the patient, confining him much less than any other apparatus, besides its cleanliness and convenience in every respect. In the remainder, I removed all the fragments of broken bone from the wound, by making an incision into the wound sufficiently large to admit of the removal of the larger fragments, and in four of these cases turned out the ends of the bones, and with a saw and pair of forceps cut them almost, or quite, square, placing them in apposition again; with the aid of binders, boards, and a roller bound above and below the wound, was able to keep the limb in good position without counter extension, or any further dressing. All these four cases recovered with a firm union, in ten weeks from the time of injury. Of the remaining four, from which I only removed the fragments of bone, but two recovered, the other two suffered constantly from diarrhœa during their whole course of treatment, and at the end of eight weeks died from exhaustion, with but little effort at union in the fracture. However, out of the nine cases treated upon this plan, seven were successful; showing a much larger per cent. in its favor than the former method. The results in these cases, as well as several cases of exsection performed for fractures of the arm, would seem to indicate that this mode of practice is by far the most "conservative surgery," when in the great majority of cases the chances of securing union by resection are two to one of saving life by amputation—especially in the lower extremities. And the chances of securing union with or without exsection are five to one in favor of the former.

This operation is equally, if not more successful in wounds of the upper, than in those of the lower extremities, as indicated by the following cases which occurred at the battle of Prairie Grove:

Col. J. C. B., 37th Ills. Inf., aged 24 years, wounded by a Minie ball passing through the left arm from behind forwards, fracturing the humerus at the junction of the middle with the lower third. The bone was very much comminuted, crushing one inch and a half of the shaft of the bone, breaking it into some half dozen fragments, besides the soft parts were considerably lacerated, and the wound extremely painful.

Treatment—Anodynes and stimulants for twenty-four hours with water dressing to the wound, and the limb kept in as easy a position as possible, when re-action came on with some febrile symptoms and an erysipelatous appearance about the wound.

Third day—Wound considerably inflamed and arm very much swollen, skin hot, face flushed, and pulse 92. Prescribed a saline cathartic, and cold water applied constantly to the arm, with an anodyne of Morphia at bed time.

Fourth day—Arm less painful, febrile symptoms subsided, and pulse 84. Prescribed three grains Dover's Powder every four hours.

Fifth day—Symptoms still subsiding; and on the tenth day all appearance of erysipelas had passed away, and the swelling very much reduced in the arm.

On the eleventh day, assisted by Medical Director Hubbard and others, I placed him under the influence of chloroform, and made a free incision five inches in length at the wound on the posterior aspect of the arm. After removing all the fragments of broken bone from the wound, turned out the fractured ends and removed their pointed extremities with the bone forceps, which in all took away two and a quarter inches of the shaft of the bone. During the operation there was but little loss of blood, and after re-adjusting the bones in proper position placed the arm in an angular box, open at the top with an angle of 135 degrees at the elbow, extending from the hand to the shoulder, and lined with cotton on the inside so as to fit the shape of the arm, and hold it in an easy, but steady position. The piece on the outer side of the box extending from the shoulder to the elbow, made separate and fastened by hinges to the bottom of the box, so that when it was necessary to examine and dress the wound, it could be readily done by turning down the side of the box.

Some three hours after dressing the wound, and placing it in the box, I returned to my quarters, near a quarter of a mile distant, but was soon followed by a messenger in great haste, stating that the Colonel was bleeding to death. I returned immediately, and found that he had bled near three pints, and the blood still escaping from the wound at a fearful rate, but not with a jet such as would indicate that it came from a single artery of any size. (The brachial artery, of course, being avoided in the operation). I at once removed the dressing, and applied cold water to the wound, which with the aid of the atmospheric air, seemed to check the hæmorrhage for a time, but only to return with renewed force. I then made compression on the brachial artery with the thumb, but this seemed to have little effect upon it, as the hæmorrhage seemed to spring from the enlarged vessels of the collateral circulation given off about the shoulder and axilla. After this effort had failed, and the hæmorrhage still continuing at a fearful rate, I at once sent for counsel, and while the messenger was gone, filled the wound with a tampon of scraped lint, which seemed to restrain the bleeding for a few minutes, when it would well up and burst out again profuse as ever. By this time—although he was in a recumbent position—symptoms of syncope began to appear, with a pale and anxious expression in the face. At this time two surgeons arrived, who, on a moment's reflection, decided to amputate at once. But I still felt a hope that the hæmorrhage might be controlled, and would not give my consent to amputate until we had made a further effort to stop the hæmorrhage; they could suggest no other means than what had been used, and decided that amputation was imperative and should be performed immediately as he was sinking rapidly. I then sent for some liquor ferri persulphatis, which I applied by saturating large pieces of sponge and introduced into the wound, so as to completely tampon it. This had a most happy effect—in less han fi vet minutes the hæmorrhage had entirely ceased, and in forty-eight hours removed the sponges without a particle of hæmorrhage occurring afterwards. Kept the arm fixed in the box above described for five weeks, when it was removed and union discovered to be quite firm in the fracture. I then dressed the arm with splints and a roller bandage from the elbow to the shoulder—leaving a gap at the wound. He soon after went on leave of absence to Danville, Ills., and returned in five weeks to resume his duties, with his arm firmly united, and no deformity except the shortening and an impaired use of the elbow joint from contraction of the muscles; but this difficulty is being rapidly obviated by the continued use of the arm.

Lieut. D. L. H., 26th Indiana Inf., aged 27 years, received two wounds at Prairie Grove. In one, the ball entered near by and to the right of the umbilicus, penetrating the external and internal abdominal muscles to the space between the latter and the transversalis muscle, then passing downwards and backwards a distance of six inches, to exit at the under margin of ribs. This wound produced very great concussion, and in a few moments the patient was in a collapsed condition, and while being carried from the field another ball struck him on the inner and anterior aspect of the right forearm three inches below the elbow joint, passing backwards and upwards through the upper end of the ulna to exit on the posterior s[illegible], opposite the base of the olecranon process, crushing the head of the bone into small fragments, separating the olecranon and coronoid processes, allowing them to be retracted for some distance by their respective muscles. The orbicular ligament of the radius was also lacerated, so as to allow a dislocation of that bone forwards and outwards. In thirty-six hours after the injury, when re-action was well established, he was placed under the influence of chloroform, and a free incision five inches in length made longitudinally through the posterior wound, and all the loose fragments of bone removed, detaching the olecranon and coronoid processes from the tendons of the triceps and brachialis anticus muscles, then with the chain saw removed one inch and a half of the pointed end of the ulna, carrying away, in all, five inches of the upper end of the ulna including the articulation. During the operation—which required some fifteen minutes—there was but little loss of blood, (using care to avoid the radial and ulnar arteries), and the patient re-acted very readily after the influence of the chloroform had passed off, and the arm placed in an angular box similar to the one above described, excepting, that along the extent of the wound, the box was left open on the outside, so as to admit of water dressings being applied to the wound. The upper end of the box on the inner side was cut so as to fit close into the axilla, and two strips of adhesive plaster, each one and a half inches wide, placed, one on the top, and the other upon the inner side of the forearm extending up to the elbow joint, and attached by their lower free extremities to the lower end of the box at the hand; making sufficient counter-extension on the radius to reduce it, and hold it in its articulation without any further dressing. For twenty-four hours nothing unusual occurred in the wound and the patient in good spirits, when a most fearful hæmorrhage came on from the small arterial vessels opening into the wound, but by tamponing the wound with pieces of sponge saturated with Liq. ferri persulph., the hæmorrhage soon subsided, but left the patient quite faint, with considerable nausea and a disposition to vomit for some hours, and after the lapse of thirty-six hours—the sponges having been removed from the wound—hæmorrhage came on again profuse as before, but another application of the styptic soon controlled the hæmorrhage, but left the patient very pale, faint, and constant nausea, with continued efforts at vomiting, and a most intolerable thirst, but could retain nothing on his stomach for five minutes at a time. Frequently the extremities were cold, the skin colorless and pulse frequent, but very feeble. This condition continued for four days, threatening very seriously the life of the patient, although there was no more return of the hæmorrhage, and by the constant use of alcoholic stimulants, kreosote, lime water and anodynes these symptoms gradually subsided, the wound commenced granulating with a healthy suppuration, and in eight weeks from the time of the operation had quite filled up and ceased discharging. The use of the arm, however, was very much impaired at first, owing to the large cicatrix in the wound after it had healed, there was a disposition to contraction of the muscles so as to prevent flexion and extension of the forearm, as well as pronation and supination to a considerable extent. But by a constant effort at the execution of these movements, this difficulty was much obviated, and on the first of April, when I saw him last, he had acquired quite good use of the arm, so much so as to be of very great service to him, though he will probably never acquire perfect use of the limb. However, its great utility will well repay the dangers he incurred in submitting to an effort to save it.

These particular cases I have given in detail, to indicate some of the worst contingencies that are likely to occur in the treatment of cases upon this plan, and which, indeed, very rarely present so many unpleasant symptoms in a single case, as the two described. Besides these, I performed resection of the radius on two cases at the elbow joint, removing three inches of the head of the bone. Also two cases of the ulna similar to the one just described, but with no untoward symptoms, and all had an early recovery, with limbs sufficiently useful to perform any ordinary business. I had also three cases of exsection of the humerus, very similar to the one described above—but without any of the alarming symptoms attending that case—all of which recovered without any delay, and a complete union in the fracture with perfect use of the limbs, save the slight deformity from shortening.

This method of treating gunshot fractures does certainly seem to have a precedence over every other; both in saving life and preserving the usefulness of the limb. The patient is always more grateful to the surgeon who saves his limb, however much deformed it may be, than to the one who mutilated him, and prunes the limbs from his body, as is often done, just for the satlsfaction of having performed an operation, that any butcher might do. This practice has been too common in the army by bloodthirsty surgeons; which to some extent accounts for the unnecessary number of mutilated soldiers who are sent home, as well as the great list of mortality in our military hospitals.

He is the best surgeon who saves the most lives, and performs the least number of amputations. This is "Conservative Surgery."

